# Phloretic Acid Improves Metabolic Dysfunction-Associated Steatotic Liver Disease in High-Fat Diet-Fed Mice

**DOI:** 10.3390/molecules31101681

**Published:** 2026-05-15

**Authors:** Sojeong Park, HwiCheol Kim, Un Ju Jung

**Affiliations:** Department of Food Science and Nutrition, Pukyong National University, 45 Yongso-ro, Nam-gu, Busan 48513, Republic of Korea; impark-sj@naver.com (S.P.); k423897@naver.com (H.K.)

**Keywords:** phloretic acid, high-fat diet, dyslipidemia, metabolic dysfunction-associated steatotic liver disease

## Abstract

Previous studies have demonstrated that phloretic acid (PA), a phenolic compound, exerts beneficial effects on inflammation, oxidative stress, and aging. However, its effects on obesity and associated metabolic abnormalities, including dyslipidemia and metabolic dysfunction-associated steatotic liver disease (MASLD), remain unclear. To evaluate the effects of PA on these obesity-related metabolic alterations and explore the underlying mechanisms, male C57BL/6J mice were divided into three groups and fed for 10 weeks with a low-fat diet (10 kcal% fat), a high-fat diet (HFD, 60 kcal% fat), or an HFD containing 0.02% (*w*/*w*) PA. PA-supplemented mice showed no significant weight loss and fat loss. However, PA supplementation significantly reduced circulating levels of free fatty acid, triglyceride, and non-high-density lipoprotein cholesterol (HDL-C) while increasing HDL-C levels in HFD-fed mice. It also reduced hepatic lipid deposition and alleviated hepatocellular injury. These effects were accompanied by the coordinated modulation of hepatic lipid metabolism, including reduced lipogenesis and cholesterol esterification, enhanced fatty acid oxidation, and increased bile acid synthesis and excretion. Furthermore, PA attenuated hepatic oxidative stress and suppressed systemic and hepatic inflammation. These observations suggest that PA may counteract HFD-induced MASLD by modulating hepatic lipid metabolism, and that its anti-inflammatory and antioxidant effects may also contribute to these metabolic improvements.

## 1. Introduction

Lipid metabolism disorders, such as dyslipidemia and metabolic dysfunction-associated steatotic liver disease (MASLD, previously known as NAFLD), have become major global health concerns [1,2,3]. Dyslipidemia is characterized by abnormal blood lipid profiles, including increases in total cholesterol (TC), low-density lipoprotein cholesterol, and triglyceride levels, as well as a decrease in high-density lipoprotein cholesterol (HDL-C). A recent global meta-analysis reported that in adults, the prevalence of hypertriglyceridemia, hypercholesterolemia, and low HDL-C levels was estimated at 28.8%, 24.1%, and 38.4%, respectively [1]. MASLD, defined as hepatic steatosis combined with at least one cardiometabolic risk factor (e.g., dyslipidemia or obesity) [4], affects approximately 30% of adults worldwide [3]. Among modifiable risk factors, an obesogenic high-fat diet (HFD) is a major dietary contributor to dyslipidemia and MASLD [5]. Chronic HFD intake promotes hepatic lipid deposition by increasing fatty acid influx and triglyceride synthesis and by perturbing cholesterol homeostasis [6,7]. The resulting excess hepatic lipid deposition induces oxidative stress and inflammation, thereby facilitating hepatocellular injury and MASLD progression [6]. In obesity-associated insulin resistance, increased fatty acid influx, enhanced hepatic endogenous fatty acid synthesis, and diminished fatty acid β-oxidation promote very-low-density lipoprotein (VLDL) overproduction, thereby contributing to hypertriglyceridemia and atherogenic dyslipidemia [8,9]. Consistently, in clinical studies, hepatic steatosis is associated with increased VLDL secretion and an atherogenic lipoprotein profile in individuals with obesity and MASLD [10,11].

Phloretic acid (PA, 3-(4-hydroxyphenyl)propionic acid) is a natural phenolic compound found in plant sources, including apple tree leaves, olives, and peanuts, and is also generated as a metabolic product of phloretin, a natural dihydrochalcone present in many fruits and vegetables [12,13]. Structurally, PA is the hydrogenated derivative of *p*-coumaric acid and is also referred to as desaminotyrosine in tyrosine metabolism [14]. The anti-aging and antioxidant properties of PA have been demonstrated in a recent study, in which PA improved stress resistance and prolonged lifespan in *Caenorhabditis elegans* model, likely via modulation of insulin/insulin-like growth factor-1 signaling and autophagy pathways [15]. Moreover, PA has been reported to inhibit macrophage foam cell formation by promoting cholesterol efflux and suppressing oxidative stress and inflammation [16]. However, the in vivo effects and underlying mechanisms of dietary PA administration in improving obesity and metabolic disorders remain unclear. Hence, this study evaluated whether dietary supplementation with PA could ameliorate HFD-induced obesity and associated metabolic disorders in C57BL/6J mice.

## 2. Results

### 2.1. Effects of Phloretic Acid on Energy Balance, Adiposity, and Plasma Leptin Levels

**Figure 1:**
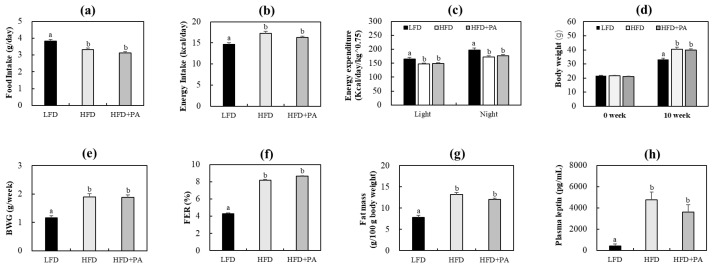
Effects of phloretic acid on food intake (**a**), energy intake (**b**), energy expenditure (**c**), body weight (**d**), body weight gain (**e**), food efficiency ratio (**f**), fat mass (**g**), and plasma leptin levels (**h**) in C57BL/6J mice fed an HFD. Data are shown as mean ± S.E. Different letters indicate significant differences among groups, as determined via one-way ANOVA followed by Duncan’s multiple range test (*p* < 0.05). LFD, low-fat diet; HFD, high-fat diet; HFD + PA, high-fat diet + phloretic acid (0.02%, *w*/*w*); BWG, body weight gain; FER, food efficiency ratio (body weight gain/food intake).

To determine whether PA affects whole-body energy balance and adiposity in HFD-fed mice, we first examined food intake, energy intake, energy expenditure, body weight, fat mass, and plasma leptin levels. Throughout the 10-week study, food intake and body weight were monitored. Relative to the low-fat diet (LFD) group, the HFD control group consumed significantly less food (Figure 1a). However, the estimated energy intake was significantly higher in the HFD group due to the higher energy density of the HFD (Figure 1b). In addition, mice fed the HFD expended significantly less energy during both light and dark phases compared with LFD-fed mice (Figure 1c). Consequently, the HFD group showed significantly greater end-point body weight, overall weight gain, food efficiency ratio, fat mass, and plasma leptin levels compared with the LFD group (Figure 1d–h). PA supplementation did not significantly alter food intake, energy intake, and energy expenditure, body weight, fat mass, or plasma leptin levels in HFD-fed mice (Figure 1). PA also did not significantly affect fasting glucose, fasting insulin, homeostasis model assessment of insulin resistance, or glucose tolerance in HFD-fed mice, although these parameters were increased in the HFD group relative to the LFD group (Supplementary Figure S1).

### 2.2. Effects of Phloretic Acid on Plasma Lipid Profiles and Bile Acid Levels in Plasma and Feces

**Figure 2:**
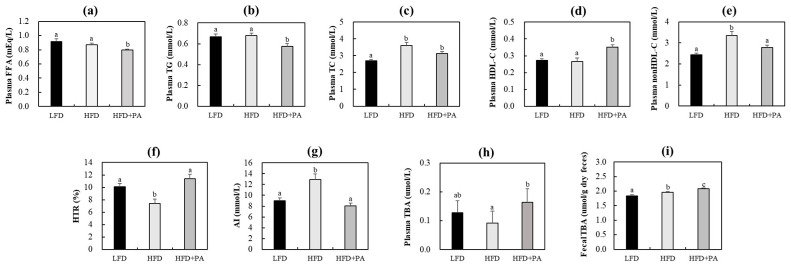
Effects of phloretic acid on plasma lipid levels (**a**–**e**), HDL-C/TC ratio (**f**), atherogenic index (**g**), and plasma and fecal total bile acid levels (**h**,**i**) in C57BL/6J mice fed an HFD. Data are shown as mean ± S.E. Different letters indicate significant differences among groups, as determined via one-way ANOVA followed by Duncan’s multiple range test (*p* < 0.05). LFD, low-fat diet; HFD, high-fat diet; HFD + PA, high-fat diet + phloretic acid (0.02%, *w*/*w*); FFA, free fatty acid; TG, triglyceride; TC, total cholesterol; HDL-C, HDL-cholesterol; non-HDL-C, non-HDL-cholesterol; HTR, HDL-C/TC ratio; AI, atherogenic index; TBA, total bile acid.

Because obesity is often associated with alterations in circulating lipid levels and bile acid metabolism, we next evaluated the effects of PA on plasma lipid parameters and bile acid levels in plasma and feces. Plasma free fatty acid (FFA), triglyceride, and HDL-C levels concentrations did not differ between the LFD and HFD groups (Figure 2a,b,d). By contrast, TC and non-HDL cholesterol (non-HDL-C) concentrations were higher in the HFD group, along with a lower HDL-C/TC ratio and a higher atherogenic index (AI) (Figure 2c,e–g). In HFD-fed mice, PA significantly decreased FFA, triglyceride, and non-HDL-C concentrations, while increasing HDL-C concentrations (Figure 2a–e). Consequently, PA supplementation significantly increased the HDL-C/TC ratio and decreased the AI (Figure 2f,g). Plasma total bile acid levels were slightly lower in the HFD group than in the LFD group, while fecal total bile acid levels were significantly increased by HFD feeding (Figure 2h,i). PA supplementation significantly increased plasma total bile acid levels relative to the HFD control group and further increased fecal total bile acid levels in HFD-fed mice. In contrast, PA supplementation did not significantly alter fecal TG levels and significantly decreased fecal TC levels compared with the HFD control group (Supplementary Figure S2).

### 2.3. Effects of Phloretic Acid on Hepatic Lipids, Liver Morphology, Plasma Aminotransferases and Inflammatory Markers, and Hepatic Oxidative Stress and Inflammation

**Figure 3:**
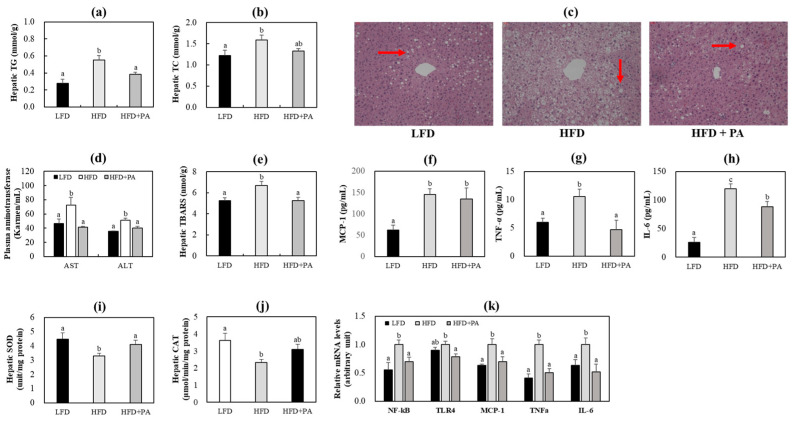
Effects of phloretic acid on hepatic lipid contents (**a**,**b**), hepatic morphology (**c**), plasma aminotransferases levels (**d**), hepatic TBARS levels (**e**), plasma inflammatory marker levels (**f**–**h**), hepatic antioxidant enzyme activity (**i**,**j**) and hepatic inflammatory genes mRNA expression (**k**) in C57BL/6J mice fed an HFD. (**a**,**b**,**d**–**k**) Data are shown as mean ± S.E. Different letters indicate significant differences among groups, as determined via one-way ANOVA followed by Duncan’s multiple range test (*p* < 0.05). (**c**) Representative histological images of liver sections stained with hematoxylin and eosin (H&E). Lipid droplets are indicated by arrows. Magnification: ×200. LFD, low-fat diet; HFD, high-fat diet; HFD + PA, high-fat diet + phloretic acid (0.02%, *w*/*w*); TG, triglyceride; TC, total cholesterol; AST, aspartate aminotransferase; ALT, alanine aminotransferase; TBARS, thiobarbituric acid reactive substances; MCP-1, monocyte chemoattractant protein-1; TNF-α, tumor necrosis factor-α; IL-6, interleukin-6; SOD, superoxide dismutase; CAT, catalase.

To determine the effects of PA on hepatic steatosis and liver injury, we examined hepatic lipid accumulation, liver morphology, plasma aminotransferases, and markers of inflammation and oxidative stress. HFD feeding induced hepatic steatosis, oxidative stress, and inflammation in mice (Figure 3). In HFD-fed mice, PA supplementation markedly lowered hepatic triglyceride and tended to lower cholesterol contents (Figure 3a,b). Moreover, PA-supplemented mice showed smaller and fewer hepatic lipid droplets and significantly lower hepatic thiobarbituric acid reactive substances (TBARS) levels, plasma aspartate aminotransferase (AST) and alanine aminotransferase (ALT) levels, and plasma tumor necrosis factor-α (TNF-α) and interleukin-6 (IL-6) levels (Figure 3c–h). Activities of hepatic antioxidant enzymes, superoxide dismutase (SOD) and catalase, were increased following PA supplementation, with SOD activity significantly increased. In addition, the HFD + PA group showed significantly decreased mRNA expression of hepatic pro-inflammatory genes, including nuclear factor kappa B (NF-κB), toll-like receptor 4 (TLR4), monocyte chemoattractant protein-1 (MCP-1), TNF-α, and IL-6 (Figure 3i–k).

### 2.4. Effects of Phloretic Acid on Hepatic Lipid Metabolism

**Figure 4:**
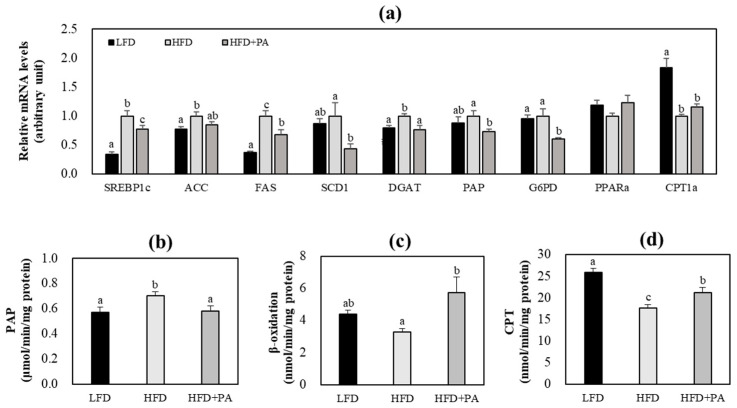
Effects of phloretic acid on hepatic fatty acid and triglyceride metabolism-related gene mRNA expression (**a**) and enzyme activity (**b**–**d**) in C57BL/6J mice fed an HFD. Data are shown as mean ± S.E. Different letters indicate significant differences among groups, as determined via one-way ANOVA followed by Duncan’s multiple range test (*p* < 0.05). LFD, low-fat diet; HFD, high-fat diet; HFD + PA, high-fat diet + phloretic acid (0.02%, *w*/*w*); SREBP1c, sterol regulatory element-binding transcription factor 1; ACC, acetyl-CoA carboxylase; FAS, fatty acid synthase; SCD1, stearoyl-CoA desaturase 1; DGAT, diacylglycerol acyltransferase; PAP, phosphatidate phosphatase; G6PD, glucose-6-phosphate dehydrogenase; PPARα, peroxisome proliferator-activated receptor-α; CPT1a, carnitine palmitoyltransferase 1A; CPT, carnitine palmitoyltransferase.

To investigate how PA may improve hepatic steatosis, we first examined the activity and activities of key enzymes and mRNA expression of genes involved in hepatic lipogenesis and fatty acid oxidation. HFD feeding increased hepatic lipogenesis and decreased fatty acid oxidation in mice, as reflected by altered mRNA expression of lipid metabolism-related genes and activities of related enzymes (Figure 4). In HFD-fed mice, PA supplementation markedly decreased the mRNA expression of hepatic lipogenic genes, including sterol regulatory element-binding transcription factor 1 (SREBP1c), fatty acid synthase (FAS), stearoyl-CoA desaturase 1 (SCD1), diacylglycerol acyltransferase (DGAT), phosphatidate phosphatase (PAP), and glucose-6-phosphate dehydrogenase (G6PD), compared with the HFD group (Figure 4a). In addition, the HFD + PA group showed significantly decreased hepatic PAP activity and increased hepatic carnitine palmitoyltransferase (CPT) and β-oxidation activities compared with the HFD control group (Figure 4b–d).

**Figure 5:**
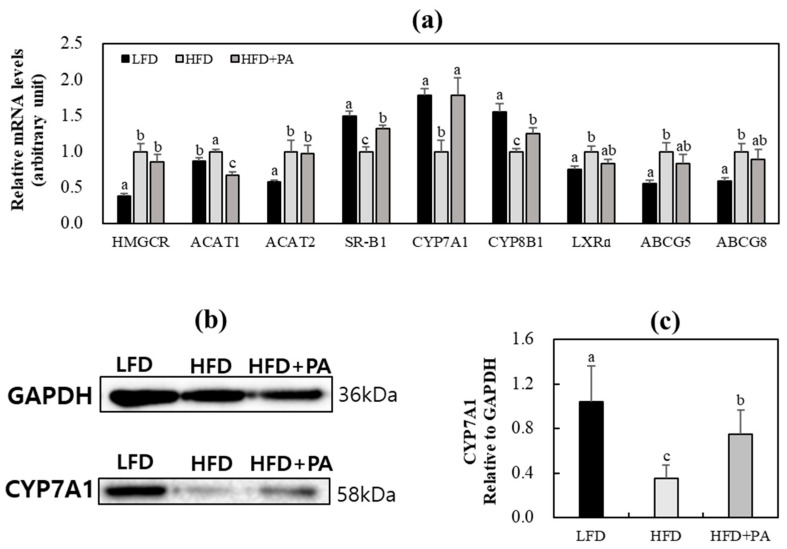
Effects of phloretic acid on hepatic cholesterol metabolism-related gene mRNA expression (**a**) and CYP7A1 protein expression (**b**,**c**) in C57BL/6J mice fed an HFD. (**a**,**c**) Data are shown as mean ± S.E. Different letters indicate significant differences among groups, as determined via one-way ANOVA followed by Duncan’s multiple range test (*p* < 0.05). (**b**) Representative Western blot analysis of CYP7A1 protein expression in liver tissue. CYP7A1 levels were normalized to GAPDH. LFD, low-fat diet; HFD, high-fat diet; HFD + PA, high-fat diet + phloretic acid (0.02%, *w*/*w*); HMGCR, 3-hydroxy-3-methylglutaryl-coenzyme A reductase; ACAT, acetyl-CoA acetyl-transferase; SR-B1, scavenger receptor class B type 1; CYP7A1, cytochrome P450 family 7 subfamily A member 1; CYP8B1, cytochrome P450 family 8 subfamily B member 1; LXRα, liver X receptor α; ABCG5, ATP-binding cassette subfamily G member 5; ABCG8, ATP-binding cassette subfamily G member 8.

Next, mRNA expression of genes involved in cholesterol metabolism was examined. Compared with the LFD group, the HFD group showed dysregulated hepatic cholesterol metabolism, as evidenced by upregulated mRNA expression of genes associated with cholesterol synthesis, esterification, and efflux/excretion, along with downregulated mRNA expression of genes involved in bile acid synthesis and composition and HDL-derived cholesterol uptake (Figure 5a). In HFD-fed mice, PA supplementation significantly downregulated hepatic acetyl-CoA acetyltransferase 1 (ACAT1) mRNA expression and markedly upregulated hepatic scavenger receptor class B type 1 (SR-B1) and cytochrome P450 family 7 subfamily A member 1 (CYP7A1), and cytochrome P450 family 8 subfamily B member 1 (CYP8B1) mRNA expression compared with the HFD control group (Figure 5a). PA supplementation also slightly attenuated HFD-induced upregulation of liver X receptor α (LXRα), ATP-binding cassette subfamily G member 5 (ABCG5), and ATP-binding cassette subfamily G member 8 (ABCG8) (Figure 5a). Hepatic CYP7A1 protein expression was also significantly lower in the HFD group than in the LFD group and was significantly increased by PA supplementation (Figure 5b,c).

## 3. Discussion

This study evaluated the influence of dietary PA supplementation on obesity-associated metabolic disturbances in HFD-fed C57BL/6J mice. PA markedly attenuated HFD-induced hepatic steatosis with no evident alterations in energy intake, energy expenditure, or body weight. These effects were accompanied by coordinated changes in markers involved in hepatic lipid metabolism, including reduced lipogenesis and cholesterol esterification, increased fatty acid oxidation, and increased bile acid synthesis and excretion. In addition, PA exerted antioxidant and anti-inflammatory effects. Collectively, these results suggest that the metabolic benefits of PA may be associated with changes in hepatic lipid metabolism and bile acid homeostasis, along with attenuation of oxidative stress and inflammation, rather than alterations in energy intake or expenditure.

In the present study, dietary PA supplementation had no marked effect on HFD-induced alterations in food intake, energy expenditure, and body weight. Although slight reductions in fat mass and plasma leptin levels were observed in PA-supplemented mice, these differences did not reach statistical significance. Leptin is an adipose tissue-derived hormone that reflects body fat mass and plays a key role in the regulation of energy balance [17]. Collectively, these results indicate that the metabolic improvements observed in PA-supplemented HFD-fed mice may not be primarily driven by changes in adiposity or overall energy balance. Similarly, several dietary phenolic compounds have been reported to improve metabolic disturbances in obese or HFD-fed animals without significant reductions in body weight or adiposity [18,19].

Dyslipidemia is a common metabolic disturbance associated with obesity and is characterized by elevated plasma triglyceride and cholesterol levels together with reduced HDL-C levels [1,20]. In the present study, HFD-fed mice did not show the characteristic features of obesity-associated dyslipidemia, as plasma FFA, triglyceride and HDL-C levels were not significantly different between the LFD and HFD groups. However, under HFD conditions, plasma TC and non-HDL-C concentrations were increased, along with a reduced HDL-C/TC ratio and an elevated AI. The HDL-C/TC ratio and AI are frequently used to evaluate cardiovascular disease risk in human studies [21,22]. Notably, PA supplementation significantly decreased plasma non-HDL-C levels while increasing HDL-C levels. Consequently, the HDL-C/TC ratio was increased and the AI was markedly reduced in PA-supplemented mice, although the physiological significance of these calculated indices in mouse models remains to be clarified because of species differences and the limited applicability of these indices to mice. PA supplementation also significantly decreased plasma FFA and triglyceride levels. However, because these lipid parameters were not significantly elevated in HFD-fed mice compared with LFD-fed mice, these changes should be interpreted as effects on basal circulating lipid levels rather than as evidence of improvement in obesity-associated dyslipidemia. Therefore, further studies using appropriate dyslipidemia models with pronounced plasma lipid abnormalities are needed to determine whether PA can improve insulin resistance- or obesity-associated plasma lipid abnormalities.

In addition, PA supplementation attenuated HFD-induced hepatic steatosis, a key pathological hallmark of MASLD characterized by excessive lipid accumulation in hepatocyte lipid droplets [23]. Consistent with previous studies, the present study demonstrated that HFD feeding markedly increased hepatic triglyceride and cholesterol levels and induced extensive lipid droplet deposition in the liver [24,25]. In contrast, PA supplementation reduced hepatic lipid accumulation and improved liver histology, as evidenced by decreased lipid droplet deposition. Moreover, PA supplementation significantly attenuated the HFD-induced increases in plasma AST and ALT levels. Elevated plasma aminotransferases, including AST and ALT, are commonly observed in patients with MASLD and are considered as markers of hepatocellular injury [26]. These findings suggest that PA may alleviate hepatic lipid accumulation and liver injury associated with MASLD.

Oxidative stress and inflammation contribute to hepatic steatosis progression [27]. In the present study, HFD feeding increased hepatic TBARS levels while inhibiting the activities of antioxidant enzymes, including SOD and catalase, in the liver. In addition, circulating inflammatory chemokine and cytokine levels (MCP-1, TNF-α, and IL-6), together with mRNA expression of hepatic inflammatory genes (NF-κB, TLR4, MCP-1, TNF-α, and IL-6), were increased in response to HFD feeding. Similarly, hepatic NF-κB mRNA and protein expression was increased in HFD-induced NAFLD models [28]. PA supplementation significantly reduced hepatic TBARS levels and restored antioxidant enzyme activities, including SOD and catalase. Furthermore, PA supplementation decreased both circulating cytokine levels and hepatic inflammatory genes mRNA expression. These findings suggest that PA may reduce hepatic oxidative stress and inflammatory responses. However, whether these changes directly contribute to the observed hepatic benefits of PA remains to be determined. In addition, further studies evaluating NF-κB phosphorylation and nuclear translocation are needed to clarify the upstream inflammatory signaling mechanisms involved [29].

Hepatic steatosis is closely linked to dyslipidemia, and dysregulation of hepatic lipid metabolism, including increased de novo lipogenesis, diminished fatty acid β-oxidation, and disturbed VLDL secretion, regarded as a major mechanism contributing to the development of both hepatic steatosis and dyslipidemia [8,9,23,30,31]. Under normal physiological conditions, de novo lipogenesis, a metabolic process that synthesizes fatty acids from non-lipid precursors, contributes approximately 10% of hepatic fatty acid input [31]. However, in obese and insulin-resistant states, this contribution can increase to approximately 40% [32]. In parallel, obesity and insulin resistance are frequently accompanied by impaired hepatic fatty acid β-oxidation, which contributes to excessive lipid deposition in the liver and promotes the development of MASLD [33,34,35]. Several key enzymes regulate hepatic lipid metabolism. Acetyl-CoA carboxylase (ACC) initiates de novo lipogenesis by catalyzing the conversion of acetyl-CoA into malonyl-CoA, an essential precursor for fatty acid synthesis and an inhibitor of CPT1a, a major enzyme controlling mitochondrial β-oxidation [36,37].

FAS and SCD1 subsequently catalyze the generation of palmitate and monounsaturated fatty acids, respectively [38,39]. PAP converts phosphatidate to diacylglycerol, which is subsequently acylated by DGAT to form triglycerides [40]. G6PD supplies NADPH required for fatty acid synthesis [41]. In the present study, HFD feeding markedly upregulated mRNA expression of hepatic lipogenic genes, including SREBP1c, ACC, FAS, and DGAT, while downregulating mRNA expression of genes implicated in fatty acid oxidation such as CPT1a. These gene expression changes were accompanied by increased hepatic PAP activity and decreased CPT activity and β-oxidation activity. Notably, PA supplementation decreased lipogenic gene mRNA expression and enzyme activity, while increasing activities of enzymes involved in fatty acid oxidation. Overall, these results suggest that PA may shift hepatic lipid metabolism from lipid synthesis toward fatty acid oxidation, which may be associated with the attenuation of hepatic lipid accumulation under HFD conditions.

In addition to its effects on fatty acid and triglyceride metabolism in the liver, PA supplementation also modulated genes involved in hepatic cholesterol metabolism. HFD feeding significantly upregulated the mRNA expression of genes associated with cholesterol synthesis and esterification, including 3-hydroxy-3-methylglutaryl-coenzyme A reductase (HMGCR), ACAT1, and acetyl-CoA acetyltransferase 2 (ACAT2), while downregulating the mRNA expression of genes implicated in bile acid synthesis and composition (CYP7A1 and CYP8B1) and HDL-derived cholesterol uptake (SR-B1) in the liver. PA supplementation significantly downregulated hepatic ACAT1 mRNA expression, although hepatic ACAT2 mRNA expression was not markedly altered. ACAT1 and ACAT2 play distinct roles in cholesterol metabolism. ACAT1 is ubiquitously expressed and primarily involved in intracellular cholesterol esterification and storage, whereas ACAT2 is mainly expressed in hepatocytes and enterocytes and contributes to cholesteryl ester formation for lipoprotein assembly [42,43]. Therefore, the downregulation of ACAT1 expression by PA supplementation may reflect decreased hepatic cholesteryl ester storage. Moreover, upregulation of hepatic CYP7A1 protein and mRNA expression following PA supplementation suggests a possible increase in hepatic cholesterol conversion into bile acids. CYP7A1 governs the rate-limiting step in cholesterol conversion into bile acids, and its upregulation suggests enhanced cholesterol catabolism [44]. The increases in plasma bile acid levels and fecal bile acid excretion observed in the PA-supplemented group further supports a possible association between PA supplementation and bile acid-related modulation of cholesterol metabolism. Although fecal TC levels were decreased in the PA-supplemented group, these findings suggest that PA-associated changes in cholesterol-related parameters may be more closely linked to bile acid metabolism than to increased fecal excretion of neutral sterols. In addition, PA supplementation restored hepatic SR-B1 mRNA expression that had been downregulated by HFD feeding. Hepatic SR-B1 mediates the hepatic selective uptake of HDL-derived cholesterol and is central to reverse cholesterol transport to the liver [45]. Taken together, changes in hepatic cholesterol-related parameters following PA supplementation may reflect, at least in part, alterations in bile acid metabolism and cholesteryl ester storage.

Based on these findings, the hepatic effects observed in PA-supplemented mice may be associated with coordinated changes in hepatic lipid metabolism, bile acid-related cholesterol metabolism, oxidative stress, and inflammatory responses. However, because the present study did not directly assess the functional contribution of bile acid metabolism, cholesteryl ester storage, antioxidant responses, or anti-inflammatory responses to the hepatic effects of PA, additional functional studies are needed to establish the causal relevance of these pathways.

Although the upstream regulators were not directly examined in this study, previous studies suggest several plausible mechanisms by which PA may modulate hepatic lipid metabolism. A recent review proposed that polyphenols can influence cholesterol metabolism by modulating CYP7A1-mediated bile acid biosynthesis and related transcriptional regulation [46]. In particular, CYP7A1 expression has been reported to be regulated primarily through the LXRα pathway. However, although PA tended to attenuate the HFD-induced increase in hepatic LXRα expression, this change was not statistically significant, suggesting that the PA-induced upregulation of CYP7A1 observed in the present study may not be explained solely by modulation of the LXRα pathway. Previous studies have suggested that CYP7A1-related bile acid biosynthesis may also be regulated through other signaling pathways, including farnesoid X receptor-, NF-κB-, and extracellular signal-regulated kinase-associated mechanisms, and may be influenced by gut microbiota-mediated bile acid deconjugation and excretion [46]. Accordingly, it is possible that multiple pathways, rather than LXRα alone, may be involved in the PA-associated changes in cholesterol metabolism. In addition, accumulating evidence has demonstrated that phloretin may ameliorate hepatic steatosis through regulation of sirtuin 1/AMP-activated protein kinase (AMPK) signaling and AMPK-dependent mitochondrial pathways [47,48] while also attenuating oxidative stress and inflammation through restoration of autophagic flux in MASLD models [49]. Because PA is a metabolite of phloretin, PA may share, at least in part, similar regulatory mechanisms, although this remains to be confirmed. Notably, previous studies on phloretin have mainly focused on lipogenesis, oxidative stress, and inflammation [47,48,49], whereas the present study suggests that PA supplementation is associated with changes in hepatic cholesterol metabolism, including increased CYP7A1 expression at both the mRNA and protein levels. Nevertheless, further studies are needed to clarify how PA regulates hepatic CYP7A1 expression and related cholesterol metabolism and whether its effects are mediated through signaling pathways similar to those reported for phloretin. In addition, transcriptomic and lipidomic analyses in future studies may provide a more comprehensive understanding of the molecular mechanisms underlying the effects of PA. Given the role of adipose tissue in MASLD-related inflammation and lipid metabolism, investigation of the molecular mechanisms of PA in adipose tissue may help clarify its metabolic actions [50,51].

Another limitation of the present study is that pharmacokinetic, tissue exposure analysis, dose-response and time-course evaluations, and formal safety evaluations of PA were not performed. Although no overt abnormal signs, reduced food intake, or abnormal body weight loss were observed in any experimental animals during the intervention, these general observations are insufficient to establish the safety of PA. Previous studies indicate that the parent compound phloretin has low oral bioavailability and that desaminotyrosine (PA) shows relatively low acute toxicity and no subchronic toxicity at low doses [52,53]. However, these findings do not establish the safety of the present intervention. Therefore, future studies should include pharmacokinetic profiling, tissue exposure analysis, dose-response and time-course evaluations, and formal safety assessment to determine the optimal effective dose and intervention period and to evaluate whether long-term PA supplementation is safe and does not cause adverse pathology.

## 4. Materials and Methods

### 4.1. Experimental Design

Male C57BL/6J mice aged 4 weeks were purchased from Hana BioTech Inc. (Pyeongtaek, Republic of Korea). The animals were kept single in stainless-steel cages and provided free access to food and water in a controlled environment maintained at 25 ± 2 °C and 50 ± 5% relative humidity under a 12 h light/dark cycle, with lights on from 07:00 to 19:00. Following a 7-day acclimation period on standard chow, mice were allocated to three experimental groups according to body weight (*n* = 8–10 per group): a LFD group (10 kcal% fat; D12450B, Research Diets, Inc., New Brunswick, NJ, USA), an HFD group (60 kcal% fat; D12492, Research Diets, Inc., USA), and an HFD group receiving 0.02% (*w*/*w*) phloretic acid (HFD + PA; H52406, Sigma-Aldrich, St. Louis, MO, USA). The assigned diets were provided for 10 weeks. The selected PA dose was based on previous studies using 0.02% (*w*/*w*) dietary phytochemical supplementation in HFD-fed C57BL/6J mice [54,55,56], and the treatment period was determined based on prior evidence that 60% HFD feeding is sufficient to induce obesity-associated metabolic phenotypes and hepatic steatosis in this model [57]. Food consumption and body weight were recorded once per week during the study period. Whole-body energy expenditure was assessed over 24 h using a metabolic cage system (OxyletPro-Physiocage, Panlab, Barcelona, Spain) 10 days before euthanasia. Fecal specimens were obtained during the last 5 days of the intervention and kept at −80 °C until analysis.

After overnight fasting at the conclusion of the experiment, mice were anesthetized with isoflurane, and blood was drawn from the inferior vena cava using EDTA-coated syringes. The liver and adipose tissues were then promptly removed, washed with saline, and weighed. Plasma and tissue specimens were preserved at −80 °C pending further analyses. All animal experiments were approved by the Animal Care and Use Committee of Pukyong National University (Approval No. 2023-55).

### 4.2. Plasma Lipids, Aminotransferases, Leptin, and Inflammatory Markers Analyses

Plasma levels of FFA, triglyceride, TC, HDL-C, AST, and ALT were quantified with commercial enzymatic assay kits (FFA: Fujifilm Wako Pure Chemical Corporation, Osaka, Japan; others: Asan Pharmaceutical Co., Seoul, Republic of Korea). The AI and HDL-C/TC ratio (HTR) were calculated as follows:

AI = (TC − HDL-C)/HDL-C

HTR (%) = (HDL-C/TC) × 100

Plasma levels of leptin and inflammatory markers (MCP-1, TNF-α, and IL-6) were measured using a mouse metabolic hormone expanded panel (Merck Millipore, Burlington, MA, USA).

### 4.3. Hepatic Lipids and TBARS Analyses

Hepatic lipids were extracted using a modified protocol based on a prior report [58]. Liver tissues were homogenized in a chloroform-methanol mixture (2:1, *v*/*v*), after which the homogenate was passed through a filter. The collected filtrate was evaporated under nitrogen at 60 °C. The dried lipids were reconstituted in FM solution. Aliquots for triglyceride and TC analysis were evaporated again and dissolved in ethanol. Hepatic triglyceride and TC concentrations were analyzed with the same kits used to assess plasma lipids.

Hepatic lipid peroxidation was assessed using a protocol adapted from Ohkawa et al. [59]. Liver samples were homogenized in 0.1 M potassium phosphate buffer (pH 7.4) using a homogenizer. The homogenate was treated with 8.1% sodium dodecyl sulfate and distilled water for 5 min at room temperature, followed by the addition of 20% acetate buffer (pH 3.5) and 0.8% thiobarbituric acid. The mixture was heated at 95 °C for 1 h. After cooling to room temperature, distilled water and n-butanol–pyridine (15:1, *v*/*v*) were supplemented. The mixture was centrifuged at 3000 rpm for 15 min at 20 °C, and the absorbance of the upper layer was measured at 532 nm. Malondialdehyde standards were prepared using the same procedure as that used for plasma TBARS.

### 4.4. Plasma and Fecal Bile Acid Analysis

Plasma and fecal total bile acids were measured using a Total Bile Acid Colorimetric assay kit (Elabscience Biotechnology Inc., Houston, TX, USA), following the protocol provided by the manufacturer. For fecal samples, bile acids were extracted from 0.5 g of fecal powder using alkaline ethanol (NaOH in 90% ethanol) at 67 °C for 1 h. After phase separation, the bile acid-containing fraction was collected, extracted with FM solution, and the organic solvents were completely evaporated. The residue was reconstituted in assay buffer and subjected to the TBA assay. Total bile acid concentration of each sample was estimated based on the calibration curve and expressed as μmol/L for plasma and normalized to fecal dry weight (μmol/g dry feces).

### 4.5. Hepatic Enzyme Activity Analysis

Liver tissues were fractionated into mitochondrial, cytosolic, and microsomal fractions as previously reported [60], with slight modifications. Briefly, liver tissues (0.5 g) were homogenized in Buffer A (0.1 M triethanolamine, 0.02 M EDTA-2Na, and 0.002 M DTT) and subjected to differential centrifugation to obtain mitochondrial, cytosolic, and microsomal fractions. Activities of enzymes involved in lipid metabolism, including PAP [61], β-oxidation [62], and CPT [63], were measured using previously described methods. Activities of antioxidant enzymes, including SOD [64] and catalase [65], were determined using established methods. Enzyme activities were normalized to protein concentrations obtained through the Bradford method [66].

### 4.6. mRNA Analysis

Liver tissue samples were processed with TRIzol reagent (Invitrogen, Carlsbad, CA, USA) to obtain total RNA. The quantity and purity of RNA were evaluated on a NanoDrop spectrophotometer (Thermo Fisher Scientific, Waltham, MA, USA). For cDNA preparation, 1 µg of total RNA was reverse-transcribed with the High-Capacity cDNA Reverse Transcription Kit (Applied Biosystems, Foster City, CA, USA). Real-time RT-qPCR amplification was carried out with SYBR PCR Master Mix (Applied Biosystems, Foster City, CA, USA), and signals were recorded on a QuantStudio™ 3 Real-Time PCR System (Applied Biosystems, Foster City, CA, USA). GAPDH served as the internal reference gene, and relative mRNA abundance was obtained by applying the 2^−ΔΔCt^ method [67].

### 4.7. Western Blot Analysis

Total protein extracts from liver tissues were prepared in RIPA buffer (Thermo Fisher Scientific, Waltham, MA, USA) supplemented with a protease inhibitor (Sigma-Aldrich, St. Louis, MO, USA). Following centrifugation at 4 °C, the supernatant fractions were retained, and protein levels were estimated with a DC Protein Assay (Bio-Rad Laboratories, Inc., Hercules, CA, USA). Protein samples, each containing 30 μg, were combined with 4× Laemmli sample buffer supplemented with β-mercaptoethanol, after which they were heated for 5 min and separated via SDS-PAGE. Proteins were subsequently transferred onto nitrocellulose membranes. After blocking in 5% non-fat milk prepared in PBS-T, the membranes were exposed overnight at 4 °C to primary antibodies against CYP7A1 (ABclonal Technology Co., Ltd., Wuhan, China) and GAPDH (Cell Signaling Technology, Inc., Danvers, MA, USA). The membranes were then incubated with an HRP-conjugated secondary antibody (Invitrogen, Carlsbad, CA, USA), and immunoreactive bands were developed with SuperSignal™ West Femto substrate (Thermo Fisher Scientific, Waltham, MA, USA). Images were captured on an imaging system from Azure Biosystems (USA), and band densities were quantified using ImageJ software (version 1.54k, National Institutes of Health, Bethesda, MD, USA).

### 4.8. Histological Analysis

A portion of each liver tissue sample was promptly fixed in 10% neutral buffered formalin for histological examination. After fixation, the tissues were dehydrated and embedded in paraffin prior to hematoxylin and eosin (H&E) staining. Morphological alterations and lipid deposition were assessed from the stained sections under a light microscope (Eclipse E200; Nikon, Tokyo, Japan) at ×200 magnification.

### 4.9. Statistical Analysis

Values are reported as mean ± standard error (S.E.). Statistical analysis was performed by one-way analysis of variance (ANOVA) followed by Duncan’s multiple range test. All analyses were conducted using IBM SPSS Statistics software (version 27.0; IBM Corp., Armonk, NY, USA). Statistical significance was assigned at *p* < 0.05.

## 5. Conclusions

The present study demonstrated that dietary PA supplementation ameliorated MASLD in HFD-induced obese mice and that the beneficial effects of PA were associated with suppression of hepatic lipogenesis, enhancement of fatty acid oxidation, stimulation of bile acid synthesis and excretion, and attenuation of oxidative stress and inflammatory responses. These findings provide new insights into the metabolic regulatory effects of PA and suggest that PA may serve as a promising dietary compound for improving obesity-associated MASLD. However, it remains unclear how the multiple pathways altered by PA, including lipid and bile acid metabolism, oxidative stress, and inflammatory responses, directly contribute to its metabolic benefits. Therefore, further studies are needed to clarify the precise mechanisms linking PA supplementation to these metabolic effects.

## Data Availability

The original contributions presented in this study are included in the article. Further inquiries can be directed to the corresponding author.

## Supplementary Materials

The following supporting information can be downloaded at: https://www.mdpi.com/article/10.3390/molecules31101681/s1, Supplementary Figure S1. Effects of phloretic acid on fasting glucose (**a**), insulin (**b**), HOMA-IR (**c**), and IPGTT (**d**) in C57BL/6J mice fed an HFD. Data are shown as mean ± S.E. Different letters indicate significant differences among groups, as determined by one-way ANOVA followed by Duncan’s multiple range test (*p* < 0.05). LFD, low-fat diet; HFD, high-fat diet; HFD+PA, high-fat diet + phloretic acid (0.02%, *w*/*w*); HOMA-IR, homeostatic index of insulin resistance, [Insulin (mU/L) × fasting blood glucose (mg/dL) × 0.05551]/22.5; IPGTT, intraperitoneal glucose tolerance test. Supplementary Figure S2. Effects of phloretic acid on fecal TG (**a**) and fecal TC (**b**) in C57BL/6J mice fed an HFD. Data are shown as mean ± S.E. Different letters indicate significant differences among groups, as determined by one-way ANOVA followed by Duncan’s multiple range test (*p* < 0.05). LFD, low-fat diet; HFD, high-fat diet; HFD+PA, high-fat diet + phloretic acid (0.02%, *w*/*w*); TG, triglyceride; TC, total cholesterol.

## Abbreviations

The following abbreviations are used in this manuscript:

MASLD	Metabolic dysfunction-associated steatotic liver disease
TC	Total cholesterol
HDL-C	High-density lipoprotein cholesterol
HFD	High-fat diet
VLDL	Very-low-density lipoprotein
PA	Phloretic acid
LFD	Low-fat diet
FFA	Free fatty acid
Non-HDL-C	Non-HDL cholesterol
AI	Atherogenic index
AST	Aspartate aminotransferase
ALT	Alanine aminotransferase
TBARS	Thiobarbituric acid reactive substances
NF-κB	Nuclear factor kappa B
TLR4	Toll-like receptor 4
MCP-1	Monocyte chemoattractant protein-1
TNF-α	Tumor necrosis factor-α
IL-6	Interleukin-6
SOD	Superoxide dismutase
SREBP1c	Sterol regulatory element-binding transcription factor 1
ACC	Acetyl-CoA carboxylase
FAS	Fatty acid synthase
DGAT	Diacylglycerol acyltransferase
SCD1	Stearoyl-CoA desaturase 1
PAP	Phosphatidate phosphatase
CPT	Carnitine palmitoyltransferase
G6PD	Glucose-6-phosphate dehydrogenase
LXRα	Liver X receptor α
HMGCR	3-Hydroxy-3-methylglutaryl-coenzyme A reductase
ACAT1	Acetyl-CoA acetyltransferase 1
ACAT2	Acetyl-CoA acetyltransferase 2
ABCG	ATP-binding cassette subfamily G member
CYP7A1	Cytochrome P450 family 7 subfamily A member 1
CYP8B1	Cytochrome P450 family 8 subfamily B member 1
SR-B1	Scavenger receptor class B type 1
AMPK	AMP-activated protein kinase
HTR	HDL-C/TC ratio
H&E	Hematoxylin and eosin
