# Supplementary material for: Phloretic Acid Improves Metabolic Dysfunction-Associated Steatotic Liver Disease in High-Fat Diet-Fed Mice
Source: Molecules. 2026 May 15;31(10):1681. doi: 10.3390/molecules31101681 (PMC13209798; doi:10.3390/molecules31101681)
Supplement: Supplementary file 1 [file molecules-31-01681-s001.zip › molecules-4224903-supplementary.pdf]

Materials and Methods

1. Plasma glucose, insulin, homeostasis model assessment of insulin resistance and intraperitoneal glucose tolerance test

Plasma glucose levels were measured using a commercial enzymatic kit (Asan Pharmaceutical, Cat. No. AM201, Seoul, Republic of Korea), and plasma insulin levels were determined using a Mouse Metabolic Hormone Expanded Panel (MME-44K-06, Merck Millipore, Burlington, MA, USA).

The homeostasis model assessment of insulin resistance (HOMA-IR), an index of insulin resistance, was calculated using the following equation.

HOMA-IR = [fasting insulin concentration (mU/L)] × [fasting glucose concentration (mg/dL) × 0.05551] / 22.5

An intraperitoneal glucose tolerance test (IPGTT) was performed one week before sacrifice to assess glucose tolerance. After a 12 h fast, mice received an intraperitoneal injection of glucose (0.5 g/kg body weight). Tail vein blood was collected at 0, 30, 60, and 120 min after glucose administration, and blood glucose levels were measured using a glucose analyzer (ACURA PLUS, i-SENS, Seoul, Republic of Korea).

2. Fecal lipids

Feces collected over the final 5 days were dried and stored at –80 °C until analysis. The dried feces were ground into a fine powder, and 0.25 g was used for lipid extraction. The dried feces were ground into a fine powder, and 0.25 g was used for lipid extraction. Lipids were extracted with 5 mL of FM solution (chloroform:methanol, 2:1, v/v) at 4 °C for 24 h. After centrifugation at 3,000 rpm for 10 min at 4 °C, 3 mL of the supernatant was collected and evaporated under nitrogen gas at 50 °C. The resulting residue was reconstituted in 0.5 mL of FM solution. Thereafter, a 200 µL aliquot was evaporated again and dissolved in 5 mL of ethanol for subsequent analysis. Fecal TG and TC levels were determined using the same enzymatic kits used for plasma TG and TC analyses.

Results

1. Effects of phloretic acid on glucose homeostasis and insulin sensitivity

Fasting plasma glucose and insulin levels were significantly higher in the HFD group than in the LFD group (Fig. S1a,b). However, PA supplementation did not significantly alter fasting glucose or insulin levels compared with the HFD control group. In addition, HOMA-IR was significantly increased in the HFD group relative to the LFD group (Fig. S1c). No significant difference in HOMA-IR values was observed between the HFD and HFD+PA groups.

During the IPGTT, blood glucose levels at 60 and 120 min were significantly higher in the HFD group than in the LFD group (Fig. S1d). Although the HFD-PA group showed lower blood glucose levels at 120 min than the HFD group, these differences were not statistically significant.

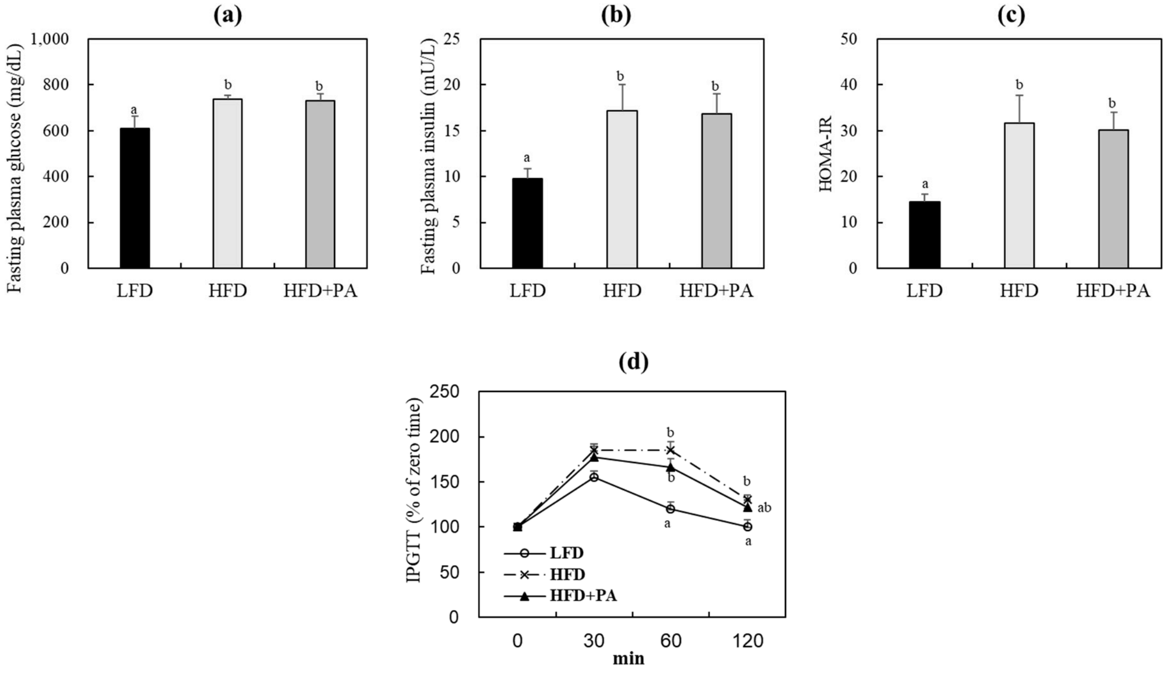

Supplementary Figure S1. Effects of phloretic acid on fasting glucose (a), insulin (b), HOMA-IR (c), and IPGTT (d) in C57BL/6J mice fed an HFD. Data are shown as mean ± S.E. Different letters indicate significant differences among groups, as determined by one-way ANOVA followed by Duncan's multiple range test ($p < 0.05$). LFD, low-fat diet; HFD, high-fat diet; HFD+PA, high-fat diet + phloretic acid (0.02%, w/w); HOMA-IR, homeostatic index of insulin resistance, [Insulin (mU/L) × fasting blood glucose (mg/dL) × 0.05551]/22.5; IPGTT, intraperitoneal glucose tolerance test.

2. Fecal lipids

The HFD group showed significantly higher fecal TG and TC levels than the LFD group (Fig. S2). PA supplementation did not significantly alter fecal TG levels compared with the HFD control group, whereas fecal TC levels were significantly decreased in the HFD+PA group.

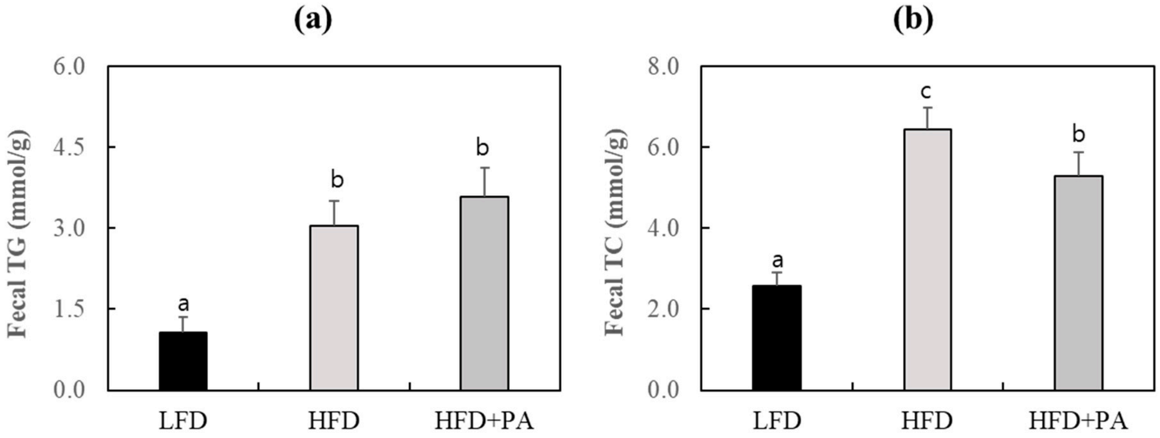

Supplementary Figure S2. Effects of phloretic acid on fecal TG (a) and fecal TC (b) in C57BL/6J mice fed an HFD. Data are shown as mean ± S.E. Different letters indicate significant differences among groups, as determined by one-way ANOVA followed by Duncan's multiple range test ($p < 0.05$). LFD, low-fat diet; HFD, high-fat diet; HFD+PA, high-fat diet + phloretic acid (0.02%, w/w); TG, triglyceride; TC, total cholesterol.
